# Modulating cell response on cellulose surfaces; tunable attachment and scaffold mechanics

**DOI:** 10.1007/s10570-017-1612-3

**Published:** 2017-12-19

**Authors:** James C. Courtenay, Christoph Deneke, Evandro M. Lanzoni, Carlos A. Costa, Yongho Bae, Janet L. Scott, Ram I. Sharma

**Affiliations:** 1 Centre for Sustainable Chemical Technologies, University of Bath, Bath, BA2 7AY UK; 2 Department of Chemistry, University of Bath, Bath, BA2 7AY UK; 3 Department of Chemical Engineering, University of Bath, Bath, BA2 7AY UK; 4 National Nanotechnology Laboratory, Centre for National Research in Energy and Materials, Campinas, São Paulo Brazil; 5 Department of Pathology and Anatomical Sciences, Jacobs School of Medicine and Biomedical Sciences, University at Buffalo, The State University of New York, Buffalo, NY 14214 USA

**Keywords:** Tunable tissue scaffold, Cellulose, Cell response, Chemical modification, Simple manufacture

## Abstract

Combining surface chemical modification of cellulose to introduce positively charged trimethylammonium groups by reaction with glycidyltrimethylammonium chloride (GTMAC) allowed for direct attachment of mammalian MG-63 cells, without addition of protein modifiers, or ligands. Very small increases in the surface charge resulted in significant increases in cell attachment: at a degree of substitution (DS) of only 1.4%, MG-63 cell attachment was > 90% compared to tissue culture plastic, whereas minimal attachment occurred on unmodified cellulose. Cell attachment plateaued above DS of ca. 1.85% reflecting a similar trend in surface charge, as determined from ζ-potential measurements and capacitance coupling (electric force microscopy). Cellulose film stiffness was modulated by cross linking with glyoxal (0.3–2.6% degree of crosslinking) to produce a range of materials with surface shear moduli from 76 to 448 kPa (measured using atomic force microscopy). Cell morphology on these materials could be regulated by tuning the stiffness of the scaffolds. Thus, we report tailored functionalised biomaterials based on cationic cellulose that can be tuned through surface reaction and glyoxal crosslinkin+g, to influence the attachment and morphology of cells. These scaffolds are the first steps towards materials designed to support cells and to regulate cell morphology on implanted biomaterials using only scaffold and cells, i.e. without added adhesion promoters.

**Electronic supplementary material:**

The online version of this article (10.1007/s10570-017-1612-3) contains supplementary material, which is available to authorized users.

## Introduction

The development of functional substitutes for damaged tissue and organs is an aim of tissue engineering (Dvir et al. 2011). This approach involves isolating healthy cells from the patient and expanding them in vitro, to increase their numbers (Salgado et al. 2013). Traditionally, the cultured cells are seeded onto a ligand-functionalised scaffold, with the ligands facilitating cell attachment (Agrawal et al. 2014a, b). Scaffolds provide a 3D support, often mimicking the natural extracellular matrix (ECM) of the cell, thus influencing cell behaviour and encouraging cell proliferation, differentiation and migration (Kular et al. 2014). The ECM is a structural support network that provides the ‘glue’ to bind cells together in tissue and consists of diverse proteins, sugars and other components.

Whether scaffolds are constructed from synthetic, or natural, biomaterials, they should be biocompatible, promote cell attachment and specialised cell functions, and, if to be implanted, be bioresorbable (Hollister et al. 2002; Agrawal and Ray 2001). Furthermore, a key challenge of tissue engineering is to design scaffolds that direct cells to attach or perform their phenotypic functions, which promote tissue functionality. Cellular responses to the substratum (attachment, proliferation and differentiation) are influenced by many factors including: surface charge (Courtenay et al. 2017; Sergeeva et al. 2016; Dadsetan et al. 2011), surface roughness (Biazar et al. 2011; Ranucci and Moghe 2001; Chang and Wang 2011), topology (Berti et al. 2013; Dugan et al. 2013), the presence of matrix proteins (Watanabe et al. 1993; Marklein and Burdick 2010; Schmedlen et al. 2002; Hersel et al. 2003), and porosity (Ninan et al. 2013; Gravel et al. 2006; Zaborowska et al. 2010), as well as the mechanical properties of the scaffold, such as Young’s modulus (Cao et al. 2016; Bäckdahl et al. 2006; Georges and Janmey 2005).

Cell affinity for a biomaterial is governed by cell/matrix interactions, which result from specific recognition among cell surface adhesion receptors, i.e. integrins, and extracellular matrix (ECM) proteins (e.g. fibronectin, vitronectin, and collagen) that have a cell-binding domain containing the Arg-Gly-Asp (RGD), or similar sequence (Yang et al. 2002). A traditional technique used to improve and regulate the degree of cell attachment to a synthetic scaffold, lacking such binding sites, is to coat with cell adhesive proteins, such as collagen and fibronectin (Li et al. 1996; Benoit and Anseth 2005; Patterson et al. 2010). However, this method of modification has potential disadvantages, such as control over isolation and purification—components of the modifying medium may elicit an inflammatory response and the proteins degrade over time (Hersel et al. 2003). Synthetic peptides have been developed to replace cell-binding proteins and the most commonly used peptide is RGD, which promotes integrin-cell adhesion on synthetic surfaces (Hersel et al. 2003). This can be a very effective way to facilitate cell attachment to synthetic surfaces, however, stable linking of RGD peptides to the surface is essential. In addition to proteins/peptides on surfaces, the mechanical properties of the scaffold surface govern cell-scaffold interactions. Thus, while the properties of the bulk scaffold material define the mechanical integrity of the scaffold, the mechanical properties of the material surface, to a depth of less than 1 nm, influence cell response (Agrawal et al. 2014a, b). Surface modifications of the biomaterial allow tailoring of surface properties without impact on bulk material properties. Thus, through surface modification, the native surfaces of biomaterials can be physically, or chemically, transformed with the primary goal of engineering desired surface chemistry (Ismail et al. 2007), topology (Viswanathan et al. 2016), reactivity (Ducheyne and Qui 1999), biocompatibility (Lin et al. 2015), hydrophilicity (Yang et al. 2002), and/or charge (Courtenay et al. 2017).

Cell function on the scaffold can be directly influenced by: cell and ECM interactions modulated via transmembrane receptors (Lu et al. 2012), soluble growth factors (Lieberman et al. 2002), and the mechanical properties of the biomaterial (Reddi 2003). At the cellular level, once attached to the scaffold, cells probe its elasticity as they anchor and pull on their surrounding, receiving mechanical feedback from the ECM or substrate (Discher et al. 2005). This process is known by the term mechanotransduction and is one of the mechanism by which cells convert bio-mechanical stimuli from the scaffolds to chemical cues which direct cell responses (Wang et al. 1993). Thus, when constructing a scaffold for tissue engineering, the mechanical properties of the biomaterial are critical in regulating and guiding cell response. This has important implications in clinical application, for example, directing the differentiation of mesenchymal stem cell (MSCs) to generate specific tissue using scaffolds with elasticity matching that of the desired tissue type (Engler et al. 2006).

Cellulose has previously been investigated as a potential scaffold material for tissue engineering as it is biocompatible and has tunable chemical and mechanical properties (Modulevsky et al. 2014; Sannino et al. 2009; Svensson et al. 2005; Torres et al. 2012). Furthermore, cellulose nanocrystals have been incorporated into a range of composite materials as reinforcements to produce hybrid scaffolds with stiffer mechanical properties (Kumar and Gupta 2008; Kumar et al. 2014; Kumar et al. 2017a, b). While native cellulose requires the presence of matrix ligands to facilitate cell attachment due to the lack of integrin binding sites on the substrate (Wu et al. 2003; Zou et al. 2001; Pelton 2009), chemical modification may be employed to alter surface chemistry, allowing cell attachment (Courtenay et al. 2017). Scaffolds produced from cellulose can range from hard composites blended with hydroxyapatite (Jiang et al. 2008), to soft hydrogels (Torres-Rendon et al. 2015), as well as variably crosslinked materials including oxidised cellulose crosslinked with diamines (Syverud et al. 2015), or other crosslinking agents such as glyoxal, glutaraldehyde or diisocyanates (Quero et al. 2011; Puspasari et al. 2015). It is hypothesised that changing the elastic modulus of cellulose scaffolds can influence how cells respond and spread on the surface through mechanotransduction. This can be achieved through chemical crosslinking to increase the elastic modulus (Syverud et al. 2015).

**Scheme 1:**
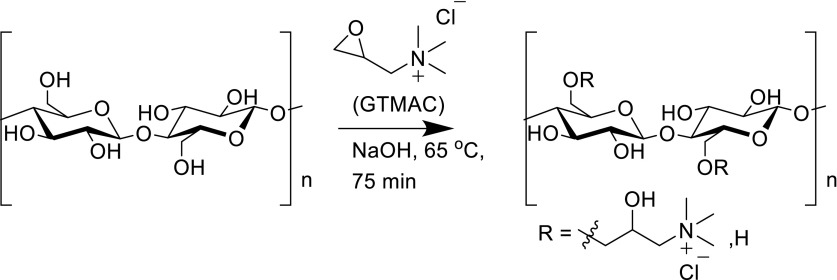
Surface derivatisation of cellulose films via the cationisation of primary OH groups accessible on the film surface by GTMAC. Cationisation results in a positive surface charge on the films

**Scheme 2:**
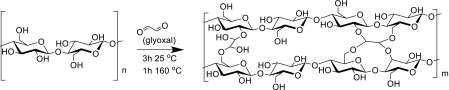
Structural modification of cellulose films through acetal, or hemiacetal, linkages formed by reaction of glyoxal with the hydroxyl groups of the cellulose, leading to increased film stiffness

Herein we describe the modulation and regulation of cellular responses through a dual approach of tuning both the chemical and mechanical properties of the cellulose-based scaffolds. Previously, we have demonstrated that surface modification, to introduce a positive surface charge to cellulose (Scheme 1), allows cell attachment in the *absence* of matrix ligands (Courtenay et al. 2017). Here we demonstrate the minimal level of surface modification required and combine this with modulation of the mechanical properties of the scaffold material, achieved by crosslinking with glyoxal (Ramires et al. 2010), which results in formation of acetal and hemiacetal linkages upon curing (Scheme 2) (Schramm and Rinderer 2000), yielding films with increased elastic moduli depending on degree of crosslinking (Quero et al. 2011).

Scaffold surfaces are probed using capacitance coupling and ζ-potential measurements to provide a sound basis for the proposed mechanism of enhanced cell attachment through complementary ionic interactions. Furthermore, changes in elastic modulus upon crosslinking are characterised for both the bulk material and the scaffold surface and the effect of the latter on cell morphology ascertained. Key surface and structural properties: surface charge and *surface* shear modulus are demonstrated to modulate cell attachment and cell spreading respectively, thus enhancing understanding of the influence of scaffold surface properties on cell responses.

## Materials and methods

Cellulose dialysis tubing (regenerated cellulose, MWCO 12,400 Da) from Sigma Aldrich was used a scaffold substrate for cell studies. For surface modifications, sodium hydroxide pellets (≥ 98%), glycidyltrimethylammonium chloride (GTMAC) (≥ 90%), 0.1 M AgNO_3_ aqueous solution (≥ 95%), indigo carmine powder (≥ 98%), and 5(6)-carboxyfluorescein (≥ 95%) were purchased from Sigma-Aldrich and used as received. For crosslinking modifications, glyoxal 40% w/w aqueous solution was purchased from Alfa Aesar and made up to required concentrations with deionised (DI) water. Aqueous solutions of AgNO_3_, NaOH and HCl, purchased from Sigma-Aldrich, were made up to the required concentrations with deionised (DI) water. Polystyrene latex beads (0.3 µm) were purchased from Sigma-Aldrich for use as tracer particles in ζ-potential measurements.

For cell studies Dulbecco’s Modified Eagle Medium (DMEM, GlutaMAX™), non-essential amino acids, sodium pyruvate, trypsin (0.05%) and trypan blue (0.4%) were purchased from Gibco and stored at 4 °C. Foetal bovine serum (FBS, non-USA origin), MG-63 cells, Pluronic F127 and formaldehyde (37% in 10–15% methanol in H_2_O solution) were purchased from Sigma-Aldrich. Phosphate buffer solution (PBS, 0.1 µm sterile filtered) was purchased from HyClone, and 6-diamidino-2-phenylindole (DAPI), phalloidin-FITC and penicillin streptomycin from Life Technologies. Norland optical adhesive 63 was purchased from Norland Products. All materials were used as received.

### Surface modification by derivitisation

Following the semi dry procedure described for modification of cellulose powder by Zaman et al. (Zaman et al. 2012), cellulose films were cationically modified with GTMAC. These GTMAC modified films are referred to as “cationic cellulose”.

Fourier Transform Infrared spectroscopy (FTIR), performed on a Perkin Elmer Spectrum 100 FTIR spectrometer, was used to confirm the presence of quaternary ammonium functional groups on cationic cellulose films. FTIR measurements were previously substantiated by ^1^H-^13^C cross polarisation/magic angle spinning NMR spectroscopy (Courtenay et al. 2017) (Figs. S1, S2, supplementary information). The degree of substitution (DS) was determined by conductometric titration (Fig. S3) against AgNO_3_(aq) solutions, conducted in triplicate.

### Structural modification by crosslinking

Cellulose dialysis membrane films, ~ 1 g, were washed thoroughly in DI water and soaked in 50 mL glyoxal solution (0.5, 1, 3, 6, or 12 wt% as required) for 3 h. The still-wet films were heated at 160 °C for 1 h and washed with copious quantities of DI water. Following this reaction, the films were cationised using the same method as previously reported (Courtenay et al. 2017) with a GTMAC:anhydroglucose unit (AGU) ratio of 2:1, and the resultant degree of substitution determined as above.

The degree of crosslinking (DXL) was determined by HPLC analysis following a method adapted from Schramm et al. (Schramm and Rinderer 2000). Briefly, dry crosslinked cellulose films (0.2–0.4 g), accurately weighed, in 20 mL 4 M NaOH were heated at 100 °C for 15 min to hydrolyse crosslinks, generating glycolic acid. The resultant solutions were filtered (PTFE, 0.45 μm disposable filter) and the concentration of glycolic acid in each solution was determined by HPLC analysis: aminex organic acid analysis column (HPX-87H, 300 mm × 7.8 mm, 50 °C), mobile phase 0.01 M H_2_SO_4_ (0.6 mL/min), and UV detector λ = 210 nm (Figs. S4, S5).

**Fig. 1:**
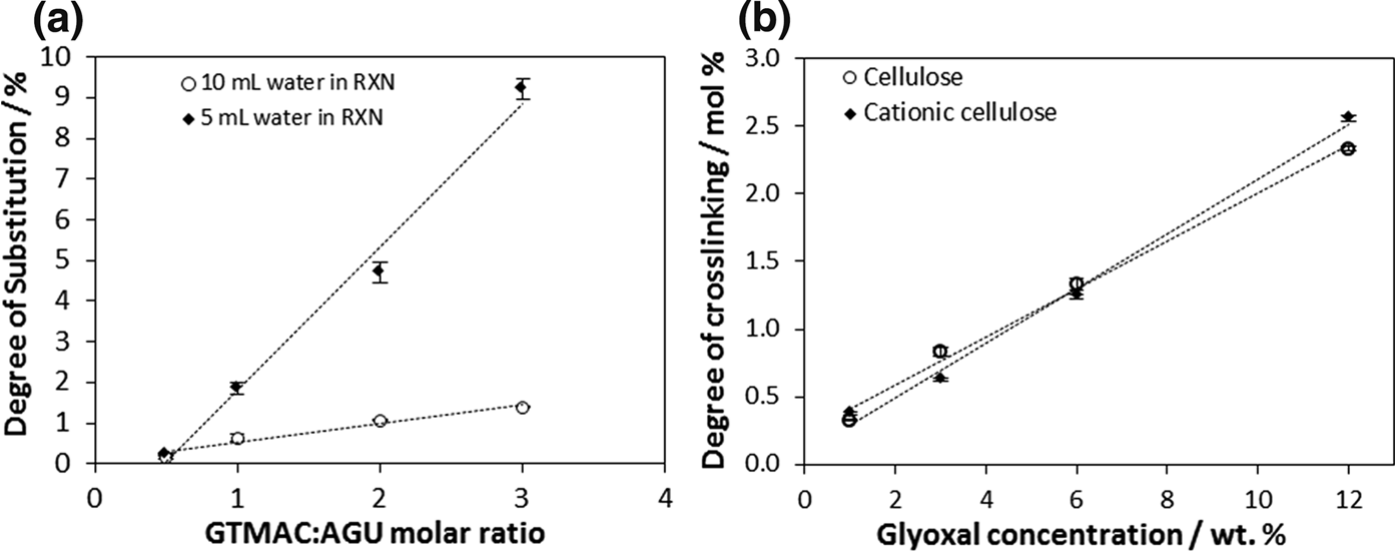
**a** DS per anhydroglucose repeat unit for the modified cellulose films determined by conductometric titration. Varying DS is achieved by using different GTMAC molar ratios and volume of water in reaction (n = 3; error bars show standard error). **b** DXL (mol%) in unmodified (R^2^ = 0.994) and cationic cellulose (R^2^ = 0.994) films determined by HPLC (n = 3, error bars show standard error)

Once the mass of glyoxal present in the crosslinked films was determined (using calibration curve, Fig. S6) the DXL was calculated using the following equation:

1 $$Degree\;of\;Crosslinking\,\% = \left[ {\frac{{162.15 \times Mol_{glyoxal} }}{{w - \left( {58.04 \times Mol_{cellulose} } \right)}}} \right]100$$

where $Mol_{glyoxal}$ is the amount of glyoxal detected by HPCL (mol), $Mol_{cellulose}$ is the amount of crosslinked cellulose present (mol) and *w* is the weight of the dried crosslinked cellulose sample (g), 162.15 is the *M*_*w*_ of the AGU and 58.04 is the difference in *M*_*w*_ between the AGU and crosslinked AGU bearing a glyoxal group. Triplicate samples were analysed for each material and an average reported (Fig. 1).

### Scaffold surface characterisation

The surface ζ-potentials of unmodified and cationic cellulose films were measured at 25 °C using a Malvern Zetasizer surface ζ-potential cell. Samples were cut to the appropriate size, mounted onto the sample plate and aligned with the laser. The measured electrophoretic mobility of 300 nm tracer particles in dispersion was recorded at varying distances from the sample surface to determine the surface ζ-potentials. Triplicate film samples were analysed for each material, the measurement repeated fifteen times per sample and an average reported.

Scanning probe microscopy was employed to obtain topography and capacitance gradient (*dC*/*dz*) images of unmodified, and cationic, cellulose films using a Park NX-10 atomic force microscope (Gouveia and Galembeck 2009; Ferreira et al. 2015). Kelvin force and capacitance coupling measurements were conducted in parallel by applying an electric AC signal at 17 kHz to the metal-coated cantilever—the DC potential was applied to the cantilever to nullify the AC signal at 17 kHz to determine the electric potential of the sample. The capacitance gradient (*dC*/*dz*), or capacitance coupling, of the tip to the sample was proportional to the second harmonic of the AC signal (34 kHz). The AFM images were processed and analysed using Gwyddion software (Necas and Klapetek 2012) and the “1D height analysis” function of the programme used to calculate the capacitance coupling signal distribution on the film.

The degree of penetration of the GTMAC reagent solution and hence the depth of penetration of modification into the bulk cellulose was evaluated by confocal florescence microscopy. Cationic films with DS of 0.6, 2.4, 4.7 and 9.2% were cut into 0.5 × 1 cm strips, washed and hydrated in 100 mL DI water, then stained by immersion in a 100 µM solution of 5(6)-carboxyfluorescein for 30 s, followed by thorough washing in DI water to remove excess dye. The films were secured to a glass slide and viewed using a LSM 510 META confocal laser scanning microscope with an EC-Plan-Neofluor 20x/0.5 PH2M27 lens. An argon laser, λ = 488 nm, was used to excite the dyed films. Multiple images acquired at 0.5 µm steps in depth were combined in a *z*-stack to determine the depth of dye penetration into the bulk of the film.

### Scaffold structural characterisation

The bulk elastic modulus of the scaffolds was determined using a Dynamic Materials Analyser (DMA1 STAR^e^ System, Mettler Toledo). The samples used were unmodified and cationic (DS = 4.7 ± 0.3%) cellulose films, with a range of crosslinking in both sets (DXL = 0–2.6%). The films (dried at 50 °C for 24 h) were cut into strips ≥ 1.50 cm in length by 0.50 cm width and the thickness recorded with a steel digital vernier micrometer calliper. The film strips were gripped between titanium tension clamp sample holders and a preload force of 1 N was applied to the sample. An offset of 10 µm was set at a frequency of 1 Hz and the elastic moduli were recorded over 5 min. To replicate “hydrated” conditions the relative humidity was set to 80% using a humidity chamber (MHG, modular humidity generator) and samples equilibrated for 10 min. Five samples were tested for each film and an average reported.

Atomic force microscopy (AFM) was used to characterise the surface shear moduli of films as previously described by Bae et al. (2014, 2016). In brief, to measure shear modulus, films were first pre-soaked in PBS overnight at room temperature. After removing PBS, cyanoacrylate adhesive was applied to glue each end of the films to the 35 mm tissue culture dish and the films re-immersed in PBS. Shear modulus was measured in force mode using a Bruker DAFM-2X BioScope AFM system. A silicon nitride probe (spring constant, 0.06 N m^−1^) with a conical tip (40 nm in diameter) was used to indent the films; 15 measurements were collected per film sample. To calculate the shear modulus, the first 600 nm of tip deflection from the horizontal was fit with the Hertz model for a cone for each measurement (Domke and Radmacher 1998). The data were analysed utilising custom MATLAB scripts kindly provided by Professor Paul Janmey.

### Cell response

Cellulose films, modified as described above, were cut into square shapes to fit a Costar^®^ tissue culture well plate, washed, and placed into wells. Loaded plates were sterilised in a Hoefer UVC 500 crosslinker for 15 min, a drop of Norland optical adhesive placed atop the sterilised films, and the films inverted and re-sterilised. Films were hydrated by adding PBS and stored at 4 °C before cell experiments.

To measure cell attachment, films were incubated with the appropriate cell culture medium for 24 h at 4 °C, the medium was removed, films seeded with MG-63 cells at a density of 10,000 cells cm^−2^ and incubated for 1 h at 37 °C. As a positive control, cells were seeded into empty tissue culture wells. At the 1 h time point, the medium was removed, cells were washed with two aliquots of PBS to remove unattached cells, remaining cells were fixed with 3.7% formaldehyde for 15 min at room temperature, washed twice with PBS, stained with DAPI for 15 min at room temperature (DAPI stains the nuclei enabling cell counts), washed with PBS and stored in PBS at 4 °C prior to image acquisition.

Cellulose films were removed from the well plate and inverted on glass microscope slides. Six independent, non-overlapping, fluorescence images of each film were acquired with a 10X objective on an EVOS optical microscope. Cell numbers were counted using ImageJ software and normalised to the area of the image. Average cell counts from the images were used to determine cell attachment by normalising to the initial seeding density, Eq. 2:

2 $$\% \,{\text{cell}}\;{\text{attachment}} = \frac{{{\text{No}}.\;{\text{of}}\;{\text{cells}}\;{\text{on}}\;{\text{scaffold}}}}{{{\text{Seeding}}\;{\text{density}}}} \times 100$$

To measure cell adhesion, seeded scaffolds, incubated for 1 h, were centrifuged at 200 rpm (8 g) for 10 min, cells were fixed, stained with DAPI and attachment determined as described above.

To measure cell morphology, films were seeded with MG-63 cells at 2500 cells cm^−2^ in serum free DMEM for 1 h to allow cells to attach without the aid of serum-containing cues. A low cell density was used to reduce cell aggregates on the surface so that individual cell morphologies could be analysed. Control experiments were performed by incubating cells in empty tissue culture well plates. The medium was then removed and replaced with serum containing medium, which is necessary for cell survival. After 24 h, cells were washed twice with PBS, fixed with formaldehyde for 15 min at room temperature, washed, permeabilised by treatment with 0.1% Triton-X for 15 min at room temperature, and washed again with PBS. Cells were stained with FITC-phalloidin (diluted 1:100) by incubating for 40 min at room temperature, washed with PBS, then stored in PBS at 4 °C prior to image acquisition. Images were acquired as described above and analysis to quantify cell area and aspect ratio was conducted using ImageJ software, as described by Fardin et al. (2010).

### Statistical analysis

An IBM SPSS Statistics Data Editor was used to perform a one-way analysis of variance (ANOVA) on data sets to determine statistically significant differences between samples at confidence levels of *p* < 0.001 (***), *p* < 0.01 (**) and *p* < 0.05 (*). Cell morphology data was presented as a box and whisker plot to convey the wide spread of cell aspect ratio and cell area.

## Results and discussion

Working towards the development of easily manufactured tissue engineering scaffold materials that are tunable to specific applications, we have demonstrated that chemical surface modification of cellulose materials, to impart positive surface charge, yielded scaffold materials that allowed ligand free attachment of cells (Courtenay et al. 2017). Here we examine the minimum level of modification required to promote cell attachment and describe the influence of scaffold surface chemistry and mechanics on the adhesion and growth of a human osteosarcoma cancer cell line, MG-63. A dual approach, utilising two easily applied chemical modifications was used to modulate the scaffold properties:

- surface charge was regulated by reaction with GTMAC to produce cationic cellulose scaffolds and
- mechanical properties of the bulk and the surface were varied by crosslinking cellulose with glyoxal.

Cellulose films, with DS ranging from 0.2 to 9.2% (determined by conductometric titration) and DXL ranging from 0.3 to 2.6% (determined by HPLC analysis), were prepared as 2D scaffolds and MG-63 cell attachment and spreading compared to unmodified films and to tissue culture polystyrene. Understanding how these facilely modified properties influence cell response aids the development of scaffolds that can promote specific, or specialised, cell function needed for proper tissue functionality and morphogenesis (Ismail et al. 2007).

### Influence of chemical modification on cell response: cell adhesion

**Fig. 2:**
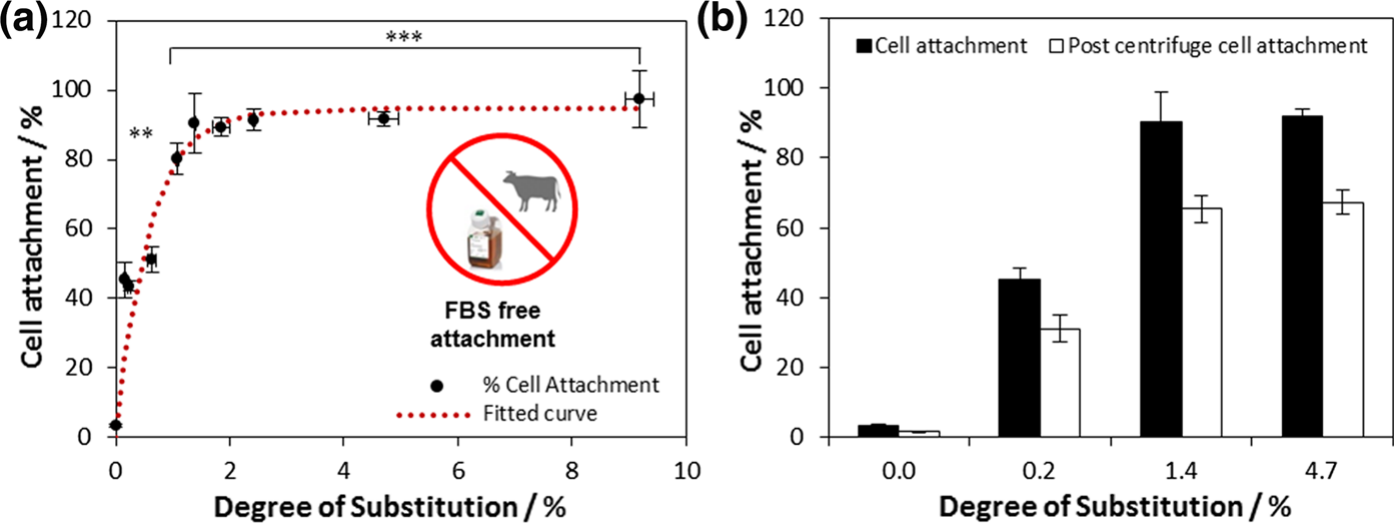
**a** The effect of varying degree of substitution on MG-63 cell attachment (after 1 h incubation at 37 °C in 5% CO_2_) on cationically modified cellulose films, with no added ligands adsorbed on the surface (n = 3; error bars show standard error). Minimal surface chemical modification resulted in significant cell attachment to cationic cellulose. Samples marked *** and ** were significantly different from unmodified cellulose with a *p* < 0.001 and *p* < 0.01 respectively. **b**) The percentage of MG-63 cells attached to modified cellulose films after centrifugation at 8*g* (n = 3 and error bars show standard error). There was no statistical difference between the cell attachment values before and post centrifugation for the modified films. The trend in increasing attachment onto films with up to ca 1.4% DS, followed by a plateau, was mirrored after the seeded scaffolds were subjected to shear, indicating good cell adhesion

Modulation of surface charge substantially enhanced cell attachment, while crosslinking had little effect (Fig. S7). Cell attachment increases with increasing DS, reaching a value of 90% (relative to tissue culture polystyrene) at DS of only ca. 1.4%—a maximum is reached between DS of 1.4 and 2% and no further enhancement in attachment follows (Fig. 2a). As expected, cells showed little affinity for unmodified cellulose, yet this very low degree of surface modification led to an amplification of cell attachment by almost *3000 times, even in the absence of any matrix proteins*, such as foetal bovine serum (FBS), added to the medium.

To test cell adhesion, seeded scaffolds were subjected to centrifugation after the initial cell attachment (1 h). Centrifugation exposes cells to shear stress, and normalising the cell counts post centrifugation to the original seeding density yields the percentage of cells remaining (Fig. 2b). Cell counts post centrifugation were consistent with cell attachment observations, suggesting that attachment was not an artefact of transient charge/charge interactions, as cells were well adhered to the positively charged cellulose, and that modified scaffolds would support adherent dependent cell behavior, such as cell spreading and protein secretion.

Previously native cellulose has been described as requiring the addition of growth factors, or matrix ligands, functionalised to the cellulose surface, to facilitate cell attachment (Wu et al. 2003; Zou et al. 2001; Pelton 2009), however, we demonstrate that a simplified two-component system (cell and biomaterial) can supersede the usual three-component system (cell, biomolecule and material) required for tissue engineering. This reduces the need to use animal (or even human) derived growth factors, potentially enabling scaffold manufacture, transport and storage and obviating some of the concerns that can arise from use of materials derived from mammalian sources, other than the intended recipient of the engineered tissue.

### Influence of chemical modification on cell response: cell morphology

Cell spreading (morphology) is an important measure of the cellular response to a given scaffold. In general, changes in cell shape from spherical to a more flattened disc-like form reflects cells encountering a scaffold surface upon which they can thrive (Lotfi et al. 2013).

**Fig. 3:**
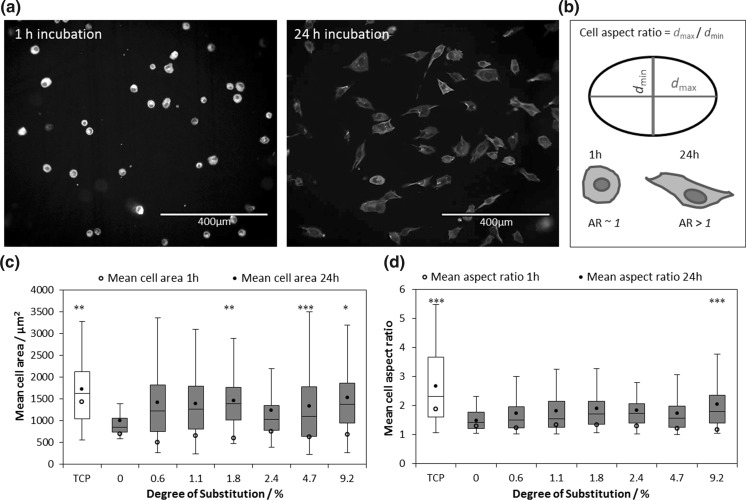
**a** Optical microscopy images of MG-63 cells spreading on cationic cellulose (9.19% DS) after incubation at 37 °C in 5% CO_2_ for 1 h (left) and 24 h (right). Attached cells were stained with DAPI (blue) and FITC-phalloidin (green) to highlight the cell nuclei and membranes respectively (scale bar = 400 µm). **b** A schematic illustrating the measurements used to determine cell aspect ratio from fluorescence images, using ImageJ software. **c**, **d** The change in cell area and aspect ratio after 24 h incubation at 37 °C in 5% CO_2_, (n = 24–435; error bars show standard error) demonstrated spreading and expansion of MG-63 occurred on the cationic cellulose scaffolds. The control scaffold was treated tissue culture plastic and cells on this surface exhibited an average area of 1725 ± 129 µm^2^ and an aspect ratio of 2.68 ± 0.17. Samples marked ***, ** and * are significantly different from unmodified cellulose with *p* < 0.001, *p* < 0.01 and *p* < 0.05 respectively. This data has also been presented in a bar graph in Fig S13. (Color figure online)

Changes in cell spreading were monitored by comparison of the projected cell area and elongation on cationised and unmodified cellulose surfaces, visualised using a fluorescent FITC-phalloidin green stain (Fig. 3a, b). For all cationised films, a significant increase in cell area (from 505–755 to 1186–1529 µm^2^) and aspect ratio (from 1.2–1.4 to 1.7–2.1) was observed after 24 h incubation at 37 °C in 5% CO_2_ (Fig. 3c, d). However, this increase was not statistically different from the cell area and aspect ratio of the very few attached cells on unmodified cellulose. This confirmed that MG-63 cells not only attached to, but also began to spread on, the cationic surface. After an initial increase in cell area due to cells flattening on the surface, the area will not necessarily increase further as cells spread out (Fig. S12). Therefore, the change in cell aspect ratio was considered to be a more relevant measure of spreading as it reflects the elongation, not the flattening, of attached cells. Interestingly, the level of cationisation of the surface did not appear to influence the cell morphology, suggesting that changes to the structural properties of the scaffold are required to further modulate spreading. This was achieved by crosslinking and we return to this discussion later. Cell area initially increases upon attachment due to the flattening of the cells, however.

### Modulation of scaffold properties: cationisation

It is hypothesised that enhanced cell attachment arose, at least in part, from a change in surface charge from negative to positive upon derivatisation and introduction of tetra-alkylammonium groups. To test this hypothesis, and to gain an understanding of the criteria for cell attachment, materials were characterised with respect to surface charge and capacitance using ζ-potential and electric force microscopy measurements. (Changes in bulk elastic modulus and surface roughness had been discounted, as no significant differences were measured between modified and unmodified materials, Figs. S9, S10).

**Fig. 4:**
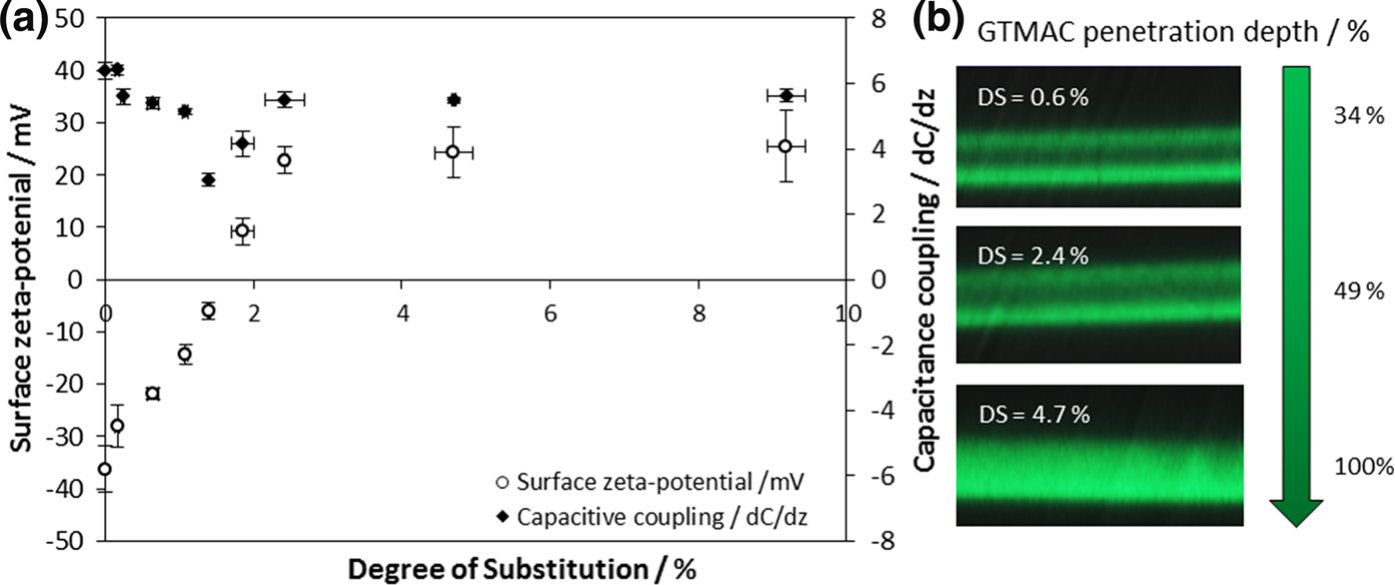
**a** ζ-potential and capacitance coupling measurements on cationic cellulose films indicated that, initially, increasing DS was correlated to increasing positive charge on the film surface, but a plateau in the surface charge properties was observed after 2.4% DS. Capacitive coupling between an EFM tip and cationic cellulose surface was generated by a 1D statistical height analysis across a 10 µm^2^ sample surface area (n = 3–5; error bars show standard error). **b** The depth of derivitisation by GTMAC was determined by staining the cationic cellulose with 5(6)-carboxyfluorescein. Constructing a z-stack from confocal microscopy images of films with varying DS revealed that the cationic derivatisation penetrates into the bulk of the cellulose after surface saturation is reached. (Color figure online)

The measured ζ-potential for unmodified cellulose films was − 36 ± 4 mV, similar to values reported previously (Hasani et al. 2008; Zaman et al. 2012), but, upon the addition of quaternary ammonium moieties, the surface ζ-potential became less negative, continuing to increase and becoming positive, 9 ± 2 mV, at 1.85% DS (Fig. 4a). Further derivatisation led to further increase in positive surface charge measured by ζ-potential, but values plateaued at 23 ± 4 mV at only 2.42% DS [reflecting values reported previously for cellulose nanocrystals (Hasani et al. 2008; Song et al. 2008)], suggesting complete saturation of available surface reactive groups. A novel observation here is that this trend is reflected in the affinity of MG-63 cells for the surface, with no further increase in numbers of cells adhered to the scaffold surface above DS of between ca. 1.8 and 2.4%.

The change in surface capacitance coupling, *dC*/*dz*, measured by electric force microscopy, supported the surface ζ-potential findings, reflecting the observed trend. This property, *dC*/*dz*, is proportional to the electric constant at the surface, however, the value measured by electric force microscopy (EFM) is independent of sign, as the instrument measures the force between the charged tip and the electrical field emanating from the sample. Therefore, surfaces of similar absolute charge density (negative or positive) would yield similar *dC*/*dz*. Unmodified cellulose has a *dC*/*dz* of 6.4 ± 0.25 AU, which, upon introduction of quaternary ammonium groups, decreases to a minimum of 3.0 ± 0.2 AU at 1.39% DS. ζ-potential shows a charge inversion (from negative to positive), which is reflected in the *dC*/*dz* values beyond this % DS. With further increased DS, measured *dC*/*dz* rises and ζ-potential continues to increase reaching 5.5 ± 0.1 AU and 23 ± 2 mV at 2.42% DS, whereafter both values plateau [in agreement with the value of 5.9 AU that we previously measured for cationic bacterial cellulose (Courtenay et al. 2017)]. Thus, it appears that, once reactive groups on the surface of the films are reacted, i.e. surface saturation is achieved, increases in measured DS reflect penetration of the GTMAC reagent into the film to greater depth, as illustrated in the confocal microscopy images (Fig. 4b).

It is instructive to consider the mechanism by which MG-63 cells adhere and the evidence suggests that favourable ionic interactions between positively charged scaffolds and the net negatively charged phospholipid groups present in the mammalian cancerous cell membrane (Song et al. 2008) are responsible for the initial “attraction” and attachment of cancer cells to the surfaces. Furthermore, using Pluronic F127 as a blocker of non-specific cell/substrate binding interactions had negligible impact on the levels of cell attachment on cationic cellulose, whereas it did reduce attachment on tissue culture plastic (Fig. S8). Moreover, MG-63 cells have been reported previously to attach onto chitosan scaffolds, which can be a positively charged polymer at some pHs (Li et al. 2005). This supports the hypothesis that cell attachment on cationic cellulose scaffolds is “surface charge driven” (Li et al. 2014; Schweizer 2009). Importantly, this response should be general (not restricted to cancerous cells or pathological state of the cell), as many cell types exhibit the same net negative charge on their plasma membranes from the phospholipids constituting the plasma membrane, so would be expected to adhere, attaching to the positively charged cellulose substrate. Studies with various cell types and pathological states are currently being investigated to explore this further.

### Modulation of scaffold properties: crosslinking

Glyoxal was chosen in this study as a chemical crosslinker due to its low toxicity to mammalian cells and ability to finely regulate the elastic moduli of the scaffolds (Ramires et al. 2010; Wang and Stegemann 2011). Both unmodified and cationic cellulose films were cured in glyoxal solutions (1–12 wt%) to achieve films with a range of crosslinking determined by HPLC (Figs. S4–S6). Quantifying the glyoxylic acid concentration post base hydrolysis enabled the DXL to be calculated. The DXL ranged from 0.3 to 2.6% (controlled by initial glyoxal concentration), with minimal difference between the starting cationic or unmodified cellulose films.

**Fig. 5:**
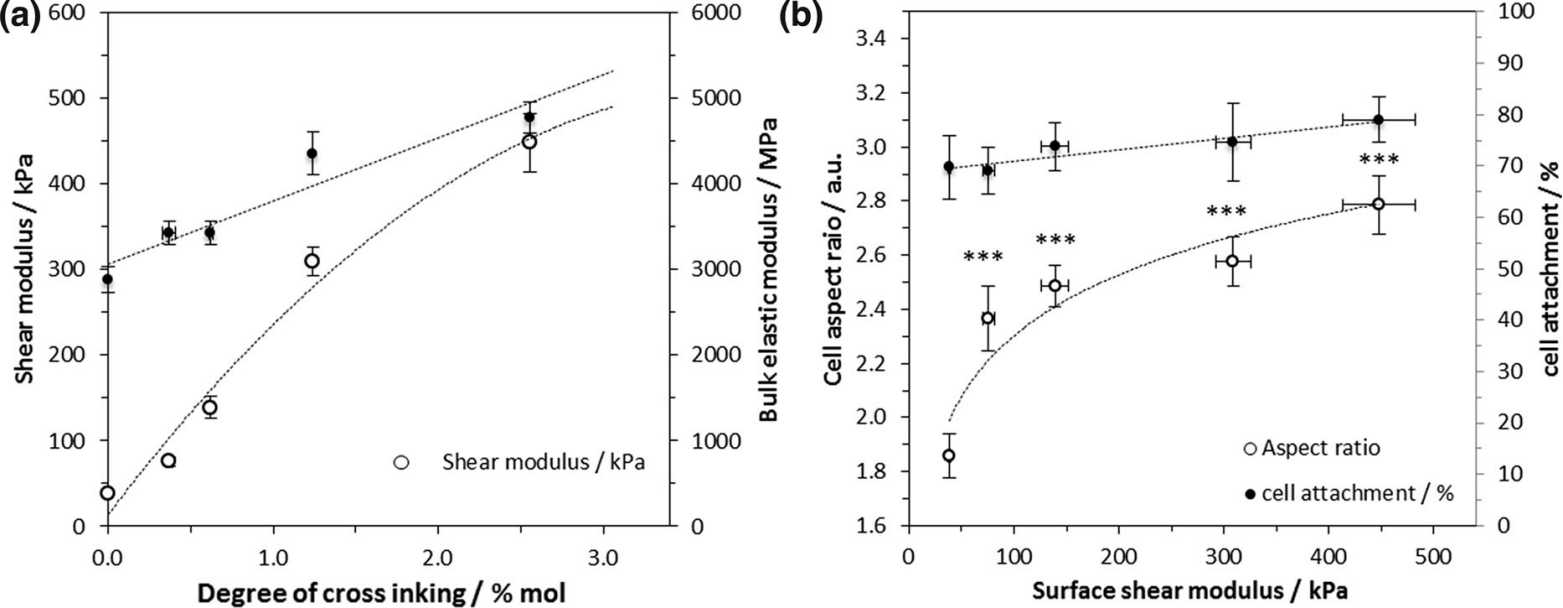
**a** Crosslinking of cationic (and unmodified) cellulose films lead to an increase in the bulk elastic modulus (measured on samples equilibrated at 80% relative humidity, n = 5, error bars show standard error, cationic cellulose, *R* ^2^ = 0.907) and surface shear modulus (n = 4; error bars show standard error, cationic cellulose, *R* ^2^ = 0.989 for data fitted to a logarithmic expression). **b** Change in MG-63 cell aspect ratio on cationic cellulose scaffolds (DS 4.7%) with increasing surface shear modulus, after 24 h incubation at 37 °C in 5% CO2 (n = 51–116, error bars show standard error). Modulating the structural properties of the scaffolds through glyoxal crosslinking had minimal effect on cell attachment, but a significant influence on the degree of cell spreading observed after 24 h incubation. Untreated cellulose scaffolds had an average cell aspect ratio of 1.86 ± 0.1, which increased significantly increased upon crosslinking. MG-63 cells, incubated on tissue culture polystyrene, were used as the control: average cell area 1725 ± 129 cm^2^ and aspect ratio 2.68. Samples marked *** were significantly different from non-crosslinked cationic cellulose with *p* < 0.001

Crosslinking of cationic cellulose films increased both bulk elastic modulus and surface shear modulus (Fig. 5a). The effect on the surface shear modulus was greater, which is significant, as the surface shear modulus more closely reflects the scaffold property defining the micro-environment at the cell-scaffold interface. The mechanical properties of the scaffolds could be tuned to further regulate cell response on the cationic scaffolds (Fig. 5b).

The bulk elastic modulus for the unmodified cellulose film (Fig. S10) was 2677 ± 195 MPa which rose to 4775 ± 182 MPa at a DXL of 2.6 mol% [comparable with values of 3917 MPa for crosslinked cellulosic materials previously reported (Retegi et al. 2010; Qi et al. 2009)]. Prior to crosslinking, cationic cellulose films exhibited elastic moduli very similar to that of unmodified cellulose, indicating that the integrity of the bulk films was not compromised by the cationisation reaction. As expected, crosslinking stiffens the cellulose films [and reduces swelling when exposed to moisture (Quero et al. 2011)], but, notably (and unexpectedly) the influence of crosslinking on the *surface shear moduli* was significantly greater than the effect on the bulk. An almost *tenfold* increase in surface shear modulus occurred upon crosslinking unmodified films (Fig. S11); from 38 ± 2 to 332 ± 37 kPa and this trend was reflected for cationised films, although the shear modulus values differed at higher degrees of crosslinking, 448 ± 35 kPa versus 332 ± 37 kPa. It is postulated that the chemical surface modification enhances crosslinking efficiency at the surface, either by more efficient reaction (with the introduced secondary alcohol beta to an ether and quaternary ammonium group), or by enhanced swelling, in the aqueous glyoxal solution, of the modified surface layer.

Cell attachment was not significantly altered upon increase in elastic, or surface shear moduli, thus, surface charge was deemed to have the greatest impact on facilitating cell attachment. However, changes in cell morphology, as measured by aspect ratio were much more dramatic.

### Influence of structural modification on cell response: cell morphology

**Fig. 6:**
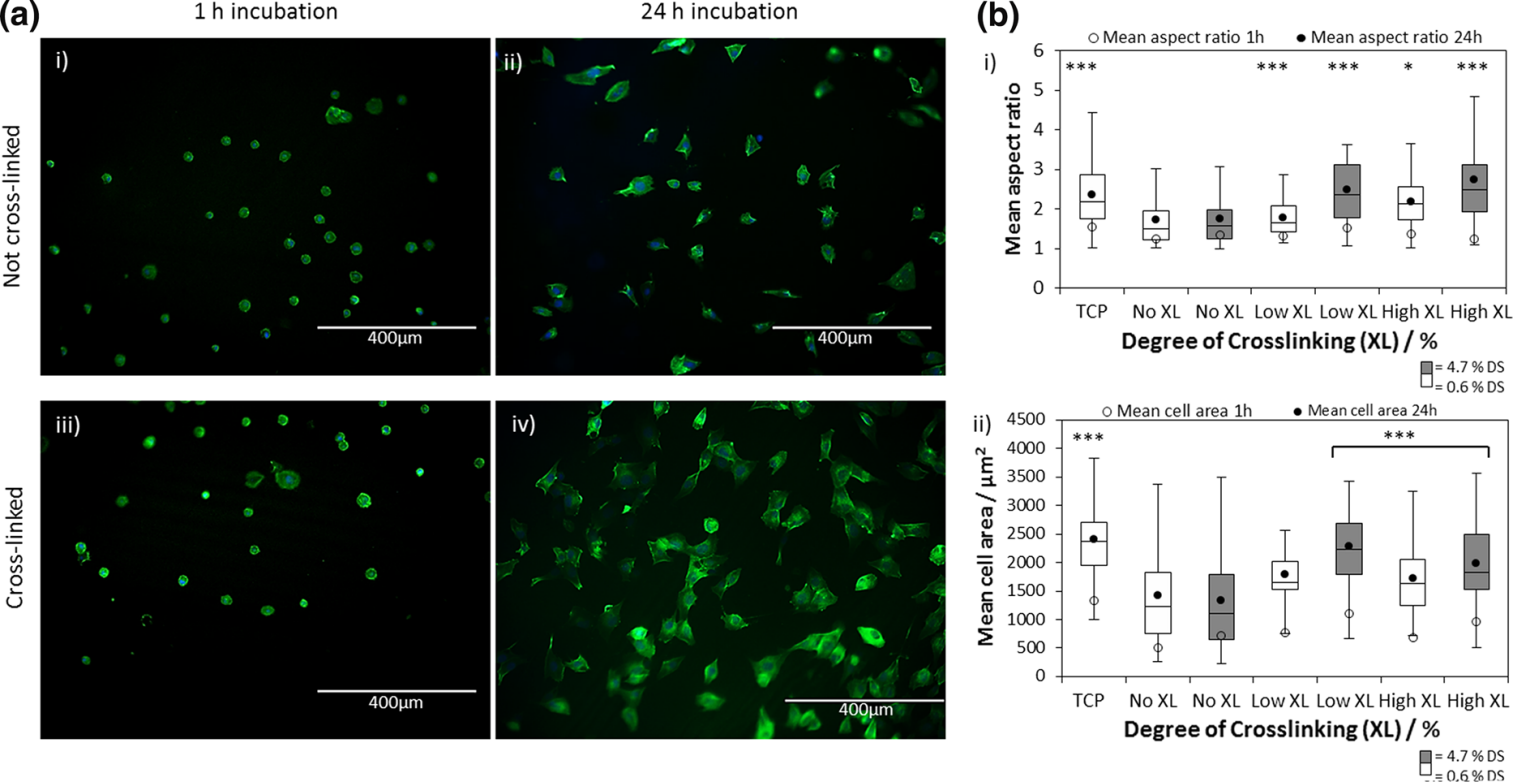
**a** Optical microscope image of cells adhered to cationic cellulose scaffold after incubation at 37 °C and 5% CO_2_: (**i**) 1 h cationic cellulose, (**ii**) 24 h cationic cellulose, (**iii**) 1 h crosslinked cationic cellulose and (**iv**) 24 h crosslinked cationic cellulose. The DS used was 4.7% and DXL was 2.6%. (The blue coloured structures are the DAPI stained nuclei and green staining, with 5(6)-carboxyfluorescein.) Scale bar = 400 µm. MG-63 cells appeared to spread out more on the stiffer cationic cellulose scaffolds crosslinked with glyoxal. **b** Influence of DS and DXL on MG-63 morphology; cell area (**i**) and aspect ratio (**ii**) on cationic cellulose scaffolds (DS 0.6 and 4.7%) treated with varying glyoxal concentrations (0, 1, 6 wt%) after 24 h incubation at 37 °C in 5% CO_2_, (n = 38–193; error bars show standard error). Cell images were analysed by ImageJ to calculate the average cell aspect ratio and area. Tissue culture plastic was used as a control, which had an area of 1725 ± 129 cm^2^ and an aspect ratio of 2.37. Samples marked ***, ** and * were significantly different from uncrosslinked cationic cellulose with *p* < 0.001, *p* < 0.01 and *p* < 0.05 respectively. This data has also been presented in a bar graph in Fig S14. (Color figure online)

The significantly greater effect of crosslinking than cationisation on cell elongation, and thus aspect ratio, is illustrated in Fig. 6a, b. Substrate stiffness has been previously reported, by Bae and co-workers, to activated FAK signalling, stimulating N-cadherin expression and increased cell spreading (Mui et al. 2015) and the effect of stiff tissue culture plastic on cell spreading is known: normal adherent cells probe elasticity as they anchor and pull on their surrounding and it has been demonstrated that, on stiffer materials, tactile sensing of the substrate by fibroblast cells feeds back on adhesion and cytoskeleton development, resulting in stronger adhesion and cell spreading (Georges and Janmey 2005; Engler et al. 2006). Therefore, modulating the scaffold mechanics can be used to further regulate cell response. It is recognised that cell response may vary from cell line to cell line, however, in this study MG-63 cells were used to probe the scaffold mechanics as they are robust yet behave in a manner similar to an osteoblast cell phenotype (Clover and Gowen 1994). Furthermore, MG-63 cells have been shown to spread on chitosan scaffolds with similar, or greater, stiffness than the cationic cellulose scaffold (Li et al. 2005). In this case it is possible to regulate cell attachment and spreading through modulating the scaffold surface charge and mechanics. Soft scaffolds do not provide enough resistance to counterbalance the tension generated by anchored MG-63 cells; as a result fewer focal adhesions are formed and cells retain their spherical shape (Georges and Janmey 2005; Engler et al. 2006). It has been suggested that MG-63 cells form stronger adhesions to stiffer scaffolds due to increased shear stress exerted on the actin fibres as they contract, resulting in a greater degree of spreading and increase cell-ECM interface area (Gumbiner 1996).

As no statistically significant effect on cell spreading was observed on moderately cationised cellulose compared to the unmodified cellulose scaffolds, crosslinking was used to stiffen the scaffolds in order to regulate cell spreading. To assess the influence of crosslinking on cell spreading, a cationised cellulose film, with low DS, was used to facilitate the cell attachment only, thus allowing the effect of crosslinking and further cationisation to be determined. The effect of increased scaffold stiffness, particularly at the surface, is important as, once cells have attached to the scaffold, responses such as: migration, proliferation and differentiation (in the case of a stem cells) are all initiated by a change in morphology of the attached cell, i.e. elongation of the cell through spreading. Thus, the ability to tune the mechanical properties of cationic cellulose scaffolds by glyoxal crosslinking in order to regulate cell response, demonstrated here, could provide advantages in clinical application, complementing approaches such as blending with hard particles (Jiang et al. 2008), or increased fibril density in bacterial cellulose (Watanabe and Yamanaka 1995; Hult et al. 2003; Bodin et al. 2006).

Thus, we have demonstrated that cellulose can form a promising and simple to modify cell scaffold material and that the combination of chemical surface modification, to introduce positive surface charge, and crosslinking, to modulate scaffold surface stiffness, provides cells with the necessary signaling required for cell attachment and spreading. It has been previously reported that the MG-63 cell line is a representative model of the osteoblast phenotype and can be used to investigate osteoblast function (Clover and Gowen 1994). The values obtained for variously crosslinked cationic cellulose, with surface shear modulus ranging from 40 to 450 kPa, suggests that these scaffolds could mimic myocytes of skeletal muscle and osteogenic environments, which have the potential to be used to generate functional musculoskeletal tissue (Janmey and Miller 2011).

Further, modulated spreading suggests opportunities in differentiation of MSCs, given their propensity to differentiate into various cell types guided by scaffold elasticity—a range of scaffold types could be devoloped to facilitate the production of different lineages, for example, soft hydrogels to rigid composites suitably mimicking brain and musculoskeletal tissue respectively (Engler et al. 2006). This offers potential advantages in:

- scaffold production (no sensitive proteinaceous components that can be prone to contamination or requiring special storage);
- scaffold use (mitigation of personal sensitivities, e.g. veganism, pertaining to use of animal derived materials); and
- clinical applications: these functionalised scaffolds could be seeded with cells and implanted into the patient without ligand pre-treatment prior to cell seeding. Once cells were adherent to the implant these could begin to produce their own extracellular matrix and would be supported by the in vivo environment.

## Conclusions

Tailored functionalised biomaterials based on cationic, crosslinked cellulose have been developed and demonstrated to support cell attachment and spreading, without the use of matrix proteins. Derivatisation of cellulose surfaces with the epoxide GTMAC—to yield positively charged cellulose surfaces—enables both attachment and spreading of cells directly on cellulose scaffolds. No added proteins, or ligands, are required. Modulated crosslinking, with glyoxal, produced materials with variable (and tunable) surface shear moduli that resulted in differential cell spreading, suggesting a simple, but effective mechanism to control response. The chemical reactions required are easily effected and the degree of both cationisation and crosslinking can be controlled. Cationisation does not compromise the integrity of the bulk material and, while crosslinking renders the bulk stiffer, the effect is greatest at the surface, thus the cell/scaffold interface can be tuned without significantly compromising the mechanical strength of the bulk construct; potentially beneficial in complex 3D scaffold constructs designed to mimic a particular organ or biological component. The elastic moduli of the crosslinked scaffolds mimicked that of myocytes and osteogenic tissue, suggesting the potential to develop such materials into tailored scaffolds to produce musculoskeletal tissue from MSCs.

Cell studies demonstrated that cell response could be further regulated by tuning the surface stiffness of the scaffolds. Thus, combining these approaches, of minimal surface modification to enable ligand-free cell attachment and modulation of mechanical properties by crosslinking, with addition of hard particles to form composites, promises to greatly extend the range of cell environments that could be mimicked.

Finally, tuning properties using cellulose as a base material and requiring only two facile chemical modifications at varying levels, offers potential advantages in production: a range of materials could be “dialed-up” and one production method could produce a range a scaffolds, or even a range of properties within one scaffold making production cost effective and enabling scale up of these two-component systems.

## Electronic supplementary material

Below is the link to the electronic supplementary material.

Supplementary material 1 (DOC 10426 kb)
