# Supplementary material for: Modulating cell response on cellulose surfaces; tunable attachment and scaffold mechanics
Source: Cellulose (Lond). 2017 Dec 19;25(2):925–40. doi: 10.1007/s10570-017-1612-3 (PMC6954015; doi:10.1007/s10570-017-1612-3)
Supplement: Supplementary file 1 — Supplementary material 1 (DOC 10426 kb) [file 10570_2017_1612_MOESM1_ESM.doc]

Supplementary Information

Modulating cell response on cellulose surfaces; tunable attachment and scaffold mechanics

*James C. Courtenay,^a,b^ Christoph Deneke,^d^* [*Evandro M. Lanzoni*](http://pubs.acs.org/action/doSearch?ContribStored=Lanzoni%2C+E+M)*,^d,e^* [*Carlos A. Costa*](http://pubs.acs.org/action/doSearch?ContribStored=Costa%2C+C+A)*,^d^ Yong Ho Bae,^f^ Janet L. Scott,^a,b*^ Ram I. Sharma^a,c*^*

**Characterisation of surface modified cellulose:**

***
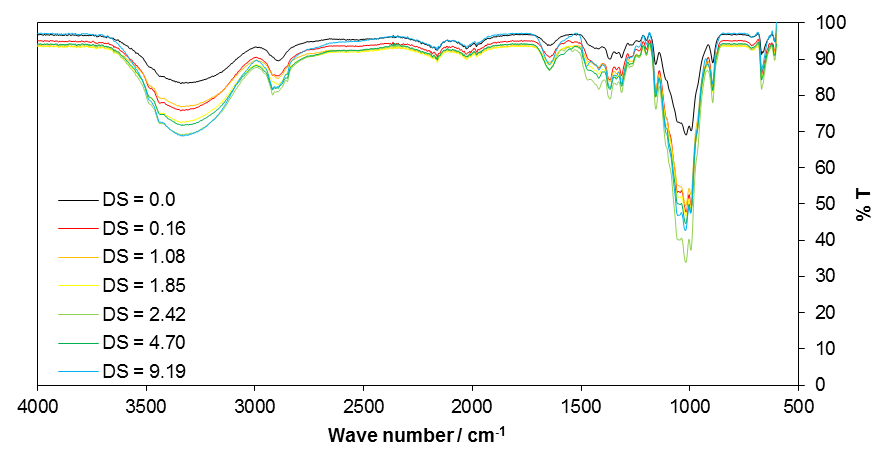
***

**Fig. S1** FTIR spectra for unmodified, cationic (DS = 0 – 9 %) cellulose films were obtained on a Perkin Elmer Spectrum 100 with a universal ATR sampling accessory; 10 scans were acquired in the range 4000 – 600 cm^-1^. FTIR: prominent bands at 1440 cm^-3^ and 1475 cm^-3^ were attributed to the CH_2_ bending mode and methyl groups of the cationic cellulose substituents in accordance with data published by Zaman *et al*.(Zaman et al. 2012)

*
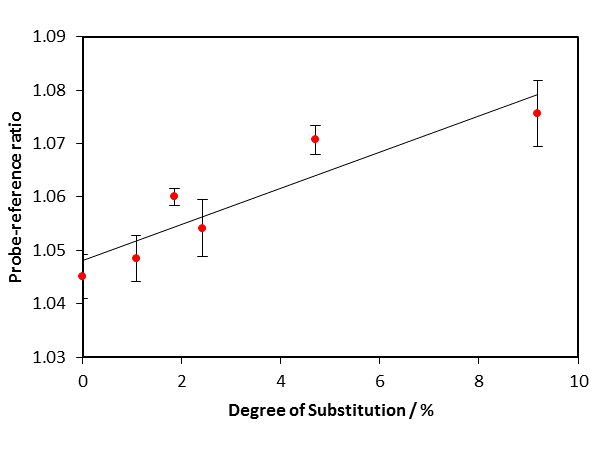
*

**Fig. S2** The degree of substitution can be correlated to the relative intensity ratio between the peak at 1475 cm^-1^ (methyl groups of the cationic group) and 2920 cm^-1^ (CH reference signal).


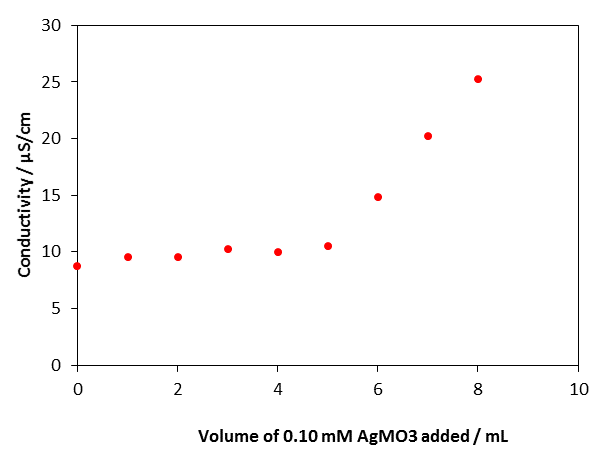


**Fig. S3** Conductometric titration curve for a cationic cellulose film in DI H_2_O titrated with 0.10 m*M* AgNO_3_ in 0.50 mL aliquots. The DS for this sample is 2.4 %.

The degree of substitution is calculated using Eq. 1:


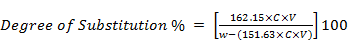
 (Eq. 1)

where *C* is the concentration of AgNO_3_ solution (*M*), *V* is the volume of AgNO_3_ solution (in dm^3^), and *w* is the weight of the dried cationic cellulose sample (g), 162.15 is the *M_w_* of the AGU and 151.63 is the difference in *M_w_* between the AGU and cationised AGU bearing a trimethylammonium chloride group.

**HPLC analysis of structural modification**


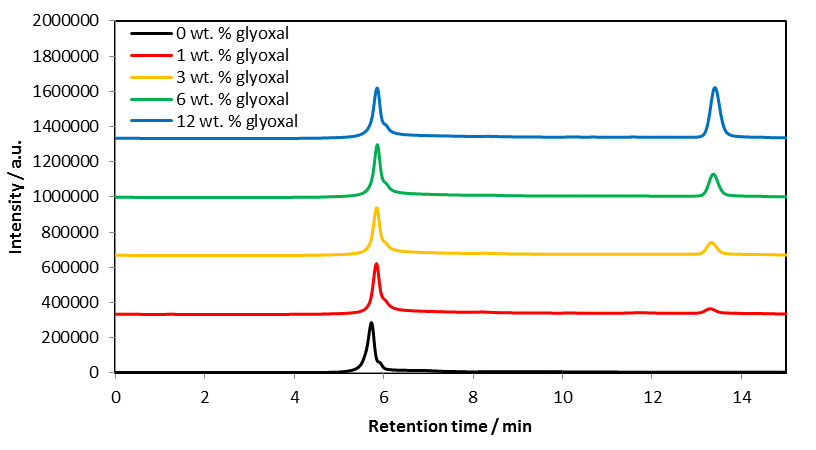


**Fig. S4** Unmodified cellulose samples. Chromatograms of the hydrolysis solutions (NaOH *c* = 4 mol L^-1^, 100 ^o^C, 15 min): A-E hydrolysis solutions for cellulose films treated with 0, 1, 3, 6, 12 wt. % glyoxal. HPLC conditions; H_2_SO_4_ = 0.01 mol L^-1^, BioRad Aminex HPX-87H; flow rate, 0.6 mL min^-1^; column oven temperature, 50 ^o^C; UV detector wavelength, 210 nm.


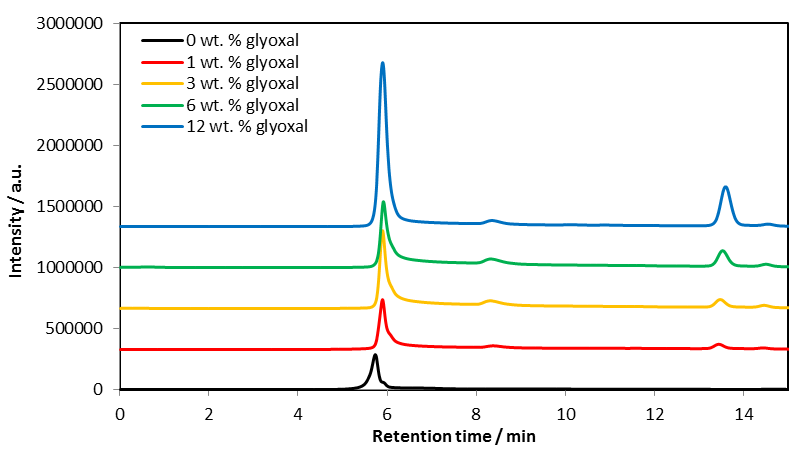


**Fig. S5** Cationic cellulose samples. Chromatograms of the hydrolysis solutions (NaOH *c* = 4 mol L^-1^, 100 ^o^C, 15 min): A-E hydrolysis solutions for cationic cellulose films treated with 0, 1, 3, 6, 12 wt. % glyoxal. HPLC conditions; H_2_SO_4_ = 0.01 mol L^-1^, BioRad Aminex HPX-87H; flow rate, 0.6 mL min^-1^; column oven temperature, 50 ^o^C; UV detector wavelength, 210 nm.


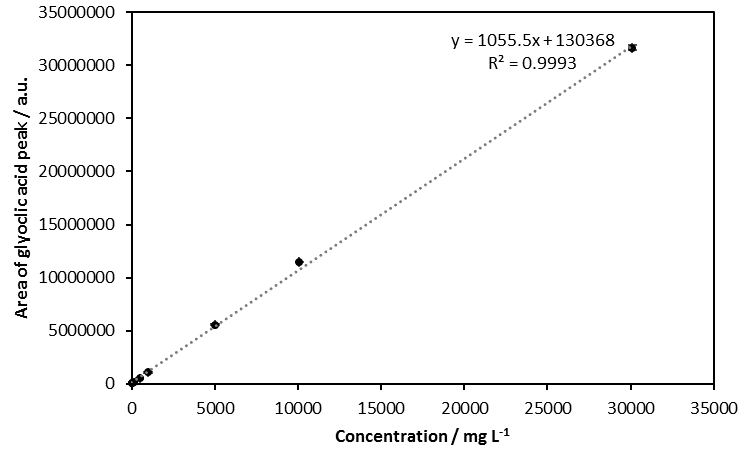


**Fig. S6** Glycolic acid peak area for prepared standard solutions (20 – 30,000 mg L^-1^). Calibration coefficient for glycolic acid was calculated from the gradient of the line to be 1055.5 a.u.2/ mg L^-1^. (n = 3, error bars = standard deviation)

**Cell attachment studies:**

**
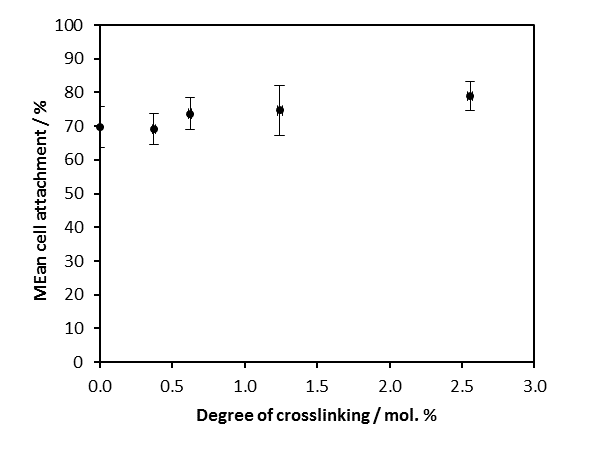
**

**Fig. S7** The influence of glyoxal crosslinking on MG-63 cell attachment. Cells were incubated 1 h at 37 ⁰C in 5 % CO_2_ on cationically modified cellulose films (DS = 4.7 %) without FBS serum ligands adsorbed on the surface (n= 3; error bars show standard error). Cell attachment on cross-linked scaffolds were not significantly different from each other

**
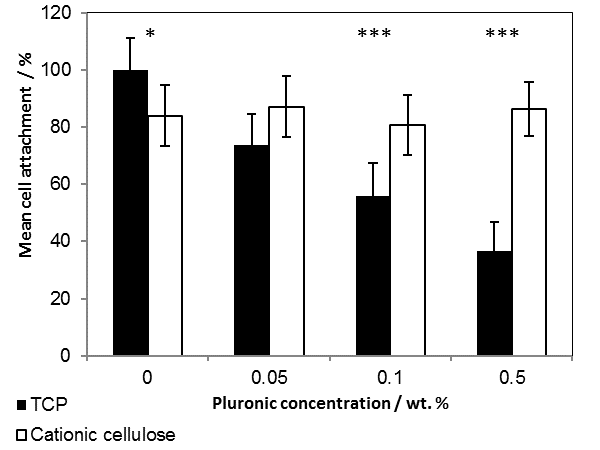
**

**Fig. S8** The effect of Pluronic L127,which blocks non-specific cell binding sites, on MG-63 cell attachment (after 1 h incubation at 37 °C in 5 % CO_2_) on tissue culture plastic and cationically modified cellulose films with no added ligands adsorbed on the surface (n=3; error bars show standard error).

Charaterisation of scaffold properties:


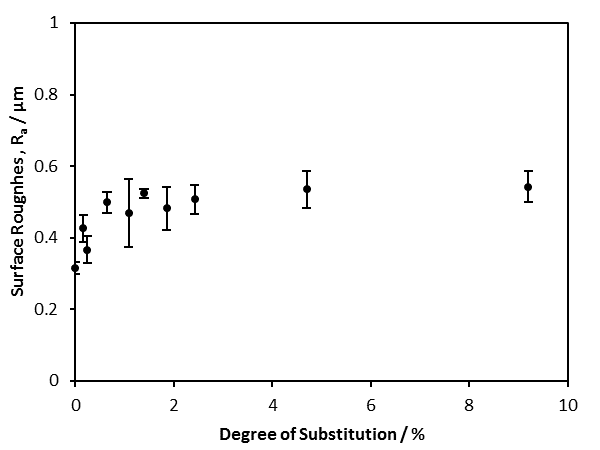


**Fig. S9** Influence of DS on scaffold surface roughness. Minimal change in surface roughness was observed on the cationically modified scaffolds. AFM images processed and analysed with Gwyddion software. The surface roughness, R_a_, was calculated using the “1D height analysis” function of the programme.


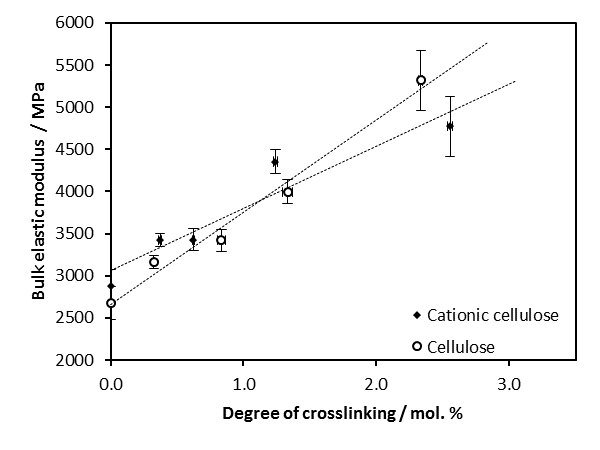


**Fig. S10** The bulk elastic modulus of hydrated cellulose films increased upon crosslinking with glyoxal. The measurements were performed at 80 % relative humidity. (n=5; error bars show standard error). Cationic cellulose, R^2^ = 0.907 & cellulose, R^2^ = 0.983.


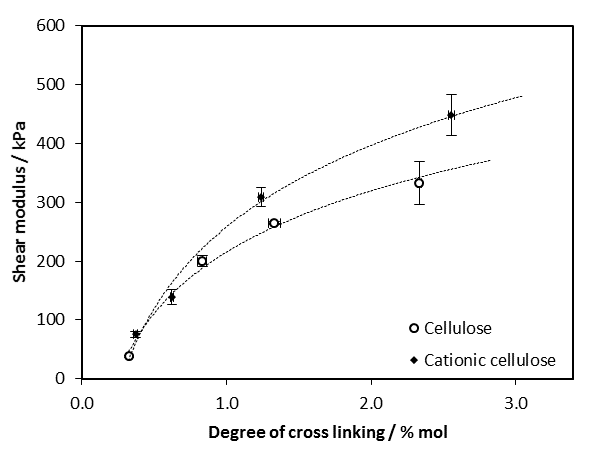


**Fig. S11** The surface shear modulus of unmodified and cationic cellulose (4.7 % DS) films with increasing amounts of crosslinking by glyoxal. Data fitted to a logarithmic expression. (n=4; error bars show standard error). Cationic cellulose, R^2^ = 0.989 & cellulose, R^2^ = 0.992.

**Cell spreading studies:**


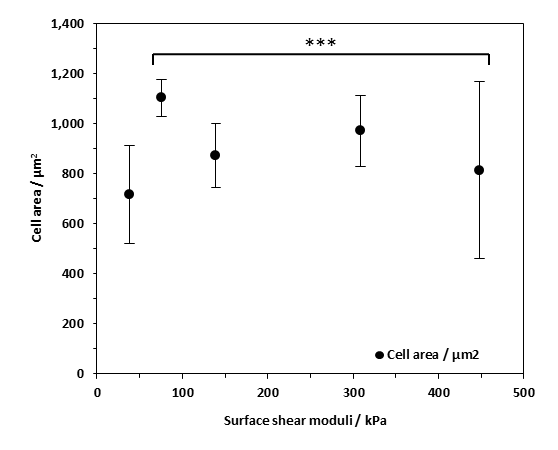


**Fig. S12** Mean cell area after 24 h incubation at 37 °C in 5 % CO_2_ on cationic cellulose scaffolds ( DS = 4.7 % with increasing degrees of cross-linking (DXL = 0 – 2.6 %). Modulating the structural properties of the scaffolds through glyoxal crosslinking did influence cell area but the relationship was not a clear one. Cell area will initially increase once attached onto the surface as they flatten. However as cells elongate the projected area will not necessarily increase. This made distinguishing the influence on greater crosslinked scaffolds difficult. Hence aspect ratio was used as a clearer measure of cell spreading. (n= 51 – 116, error bars show standard error). MG-63 cells, incubated on tissue culture polystyrene, were used as the control: average cell area 1725 ±129 cm^2^ . *** were significantly different from non-crosslinked cationic cellulose with p value < 0.001.


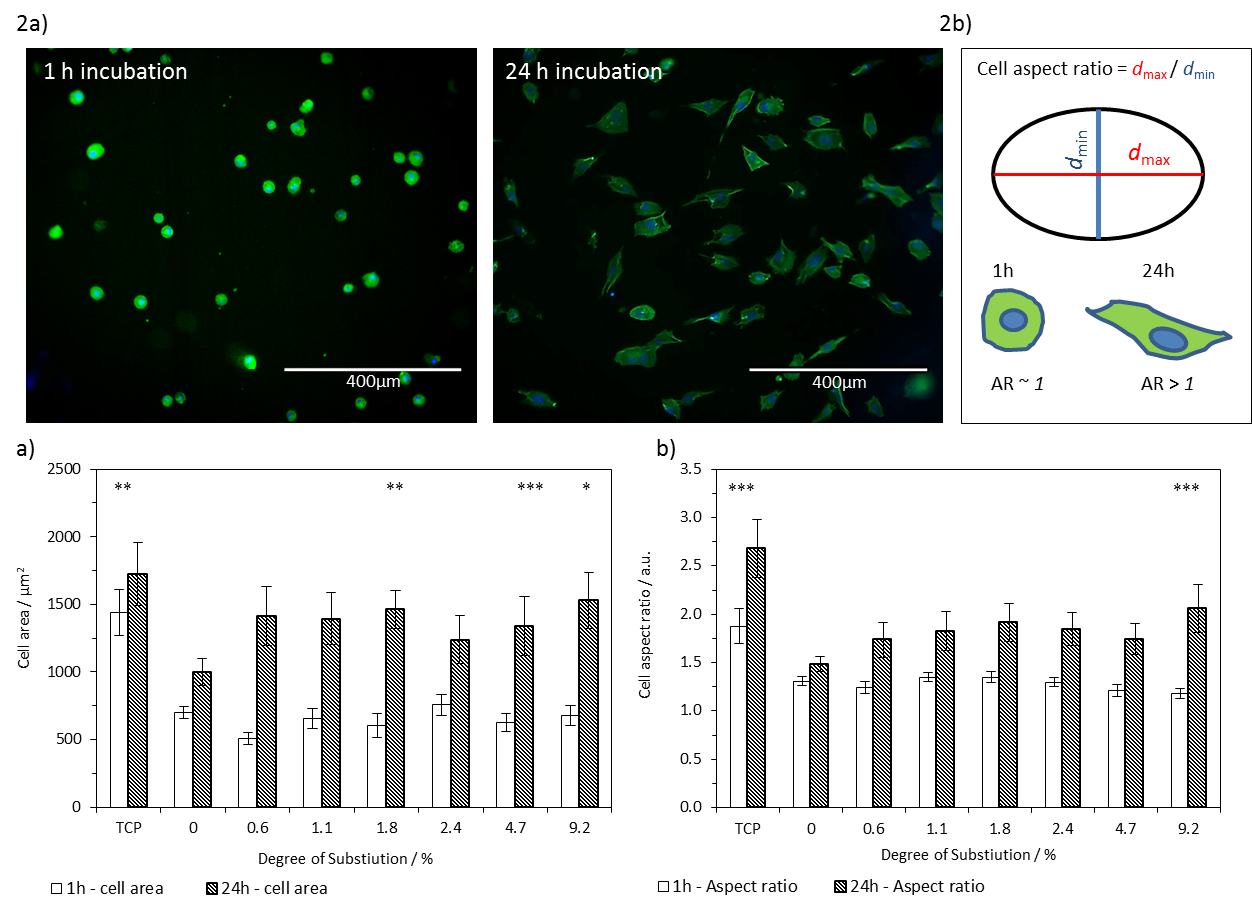


**Fig. S13 a)** The change in cell area; **b)** and aspect ratio after 24 h incubation at 37 ^o^C in 5 % CO_2_, (n= 24 - 435; error bars show standard error) demonstrated spreading of MG-63 occurred on the cationic cellulose scaffolds. The control scaffold was treated tissue culture plastic and cells on this surface exhibited an average area of 1725 ±129 µm^2^ and an aspect ratio of 2.68 ±0.17. Samples marked ***, ** and * are significantly different from unmodified cellulose with p < 0.001, p < 0.01 and p < 0.05 respectively.


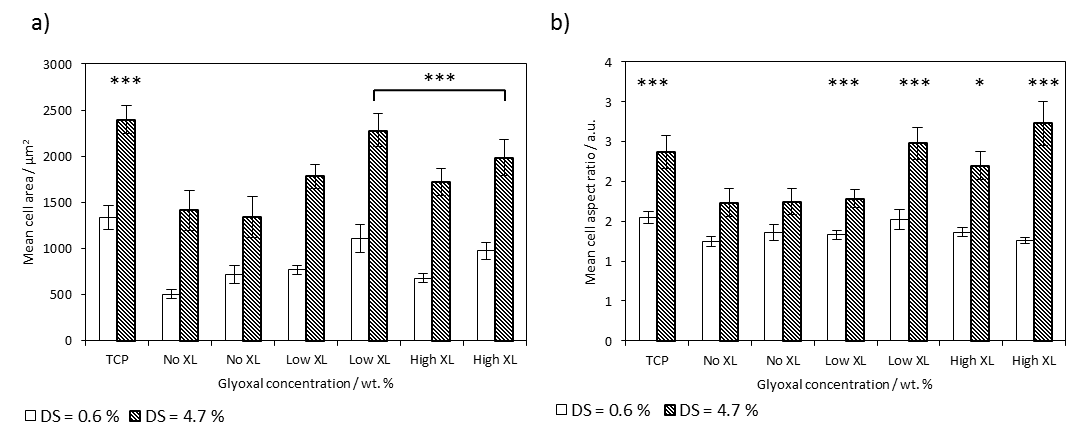


**Fig. S14** Influence of DS and DXL on MG-63 morphology; cell area (a) and aspect ratio (b) on cationic cellulose scaffolds (DS 0.6 and 4.7 %) treated with varying glyoxal concentrations ( 0, 1, 6 wt. %) after 24 h incubation at 37 ^o^C in 5 % CO_2_, (n= 38 - 193; error bars show standard error). Cell images were analysed by ImageJ to calculate the average cell aspect ratio and area. Tissue culture plastic was used as a control, where cells had an area of 1725 ±129 cm^2^ and an aspect ratio of 2.37. Samples marked ***, ** & * were significantly different from uncross-linked cationic cellulose with p < 0.001, p < 0.01 and p < 0.05 respectively.

**References:**

Zaman M, Xiao H, Chibante F, Ni Y (2012) Synthesis and characterization of cationically modified nanocrystalline cellulose. Carbohydr Polym 89:163–70. doi: 10.1016/j.carbpol.2012.02.066
